# Supplementary material for: Absolute and relative intensities of solo, free-form dancing in adults: A pilot study
Source: PLoS One. 2024 Nov 18;19(11):e0313144. doi: 10.1371/journal.pone.0313144 (PMC11573202; doi:10.1371/journal.pone.0313144)
Supplement: S1 Data — (ZIP) [file pone.0313144.s001.zip › McCullough_AbsRel_PLOSONE_Data_DataDictionary/AbsRelFreeDanceAdults_PLOS_ONE_DataDictionary.docx]

**Data Dictionary**

Study Title: Absolute and relative intensities of free-form dance among adults: A pilot study

Corresponding Author: Aston K. McCullough, PhD, MS, MA

| Variable | Description |
| --- | --- |
| METS_ModMusic | Metabolic equivalents during free-form dancing at a self-determined moderate intensity with music |
| METS_ModNoMusic | Metabolic equivalents during free-form dancing at a self-determined moderate intensity without music |
| METS_VigMusic | Metabolic equivalents during free-form dancing at a self-determined vigorous intensity with music |
| METS_VigNoMusic | Metabolic equivalents during free-form dancing at a self-determined vigorous intensity without music |
| PrHRmax_ModMusic | Percent of age-predicted maximal heart rate during free-form dancing at a self-determined moderate intensity with music |
| PrHRmax_ModNoMusic | Percent of age-predicted maximal heart rate during free-form dancing at a self-determined moderate intensity without music |
| PrHRmax_VigMusic | Percent of age-predicted maximal heart rate during free-form dancing at a self-determined vigorous intensity with music |
| PrHRmax_VigNoMusic | Percent of age-predicted maximal heart rate during free-form dancing at a self-determined vigorous intensity without music |
| PrHRR_ModMusic | Percent of heart rate reserve reached during free-form dancing at a self-determined moderate intensity with music |
| PrHRR_ModNoMusic | Percent of heart rate reserve reached during free-form dancing at a self-determined moderate intensity without music |
| PrHRR_VigMusic | Percent of heart rate reserve reached during free-form dancing at a self-determined vigorous intensity with music |
| PrHRR_VigNoMusic | Percent of heart rate reserve reached during free-form dancing at a self-determined vigorous intensity without music |
| WRRMR_ModMusic | Work rate-to-resting metabolic rate during free-form dancing at a self-determined moderate intensity with music |
| WRRMR_ModNoMusic | Work rate-to-resting metabolic rate during free-form dancing at a self-determined moderate intensity without music |
| WRRMR_VigMusic | Work rate-to-resting metabolic rate during free-form dancing at a self-determined vigorous intensity with music |
| WRRMR_VigNoMusic | Work rate-to-resting metabolic rate during free-form dancing at a self-determined vigorous intensity without music |
| RPE_ModMusic | Rating of perceived exertion during free-form dancing at a self-determined moderate intensity with music |
| RPE_ModNoMusic | Rating of perceived exertion during free-form dancing at a self-determined moderate intensity without music |
| RPE_VigMusic | Rating of perceived exertion during free-form dancing at a self-determined vigorous intensity with music |
| RPE_VigNoMusic | Rating of perceived exertion during free-form dancing at a self-determined vigorous intensity without music |
| yearsDanceExp | Self-reported years of dance training experience |
| BMI | Body mass index |
| age | Chronological age in years |
| restingHR | Resting heart rate in beats per minute |
| gender | Self-identified gender (1 = cisgender woman, 2 = cisgender man, 5 = other gender identity) |
| rmr | Resting metabolic rate in metabolic equivalents |
| rmrCV | Coefficient of variation for resting metabolic rate data |
| modMusicTempo | Tempo of self-selected music (bpm) during self-determined moderate intensity dancing |
| vigMusicTempo | Tempo of self-selected music (bpm) during self-determined vigorous intensity dancing |
| musicOrder | Randomized order of music exposure during free-form dancing (1 = music, followed by no music; 2 = no music followed by music) |
