# Supplementary material for: Absolute and relative intensities of solo, free-form dancing in adults: A pilot study
Source: PLoS One. 2024 Nov 18;19(11):e0313144. doi: 10.1371/journal.pone.0313144 (PMC11573202; doi:10.1371/journal.pone.0313144)
Supplement: Attachment — Submitted filename: PLOS_ONE_CoverLetter_AbsRelFreeFormDanceAdults_Rev07232024.pdf [file pone.0313144.s002.pdf]

Emily Chenette, PhD
Editor-in-Chief, *PLOS ONE*
PLOS
1265 Battery Street, Suite 200
San Francisco, CA 94111
United States

23 July 2024

Dear Dr. Chenette:

On behalf of my research group, I am pleased to resubmit the manuscript titled "Absolute and relative intensities of solo, free-form dancing in adults: A pilot study" to be considered for publication as an original research article in *PLOS ONE*. Thank you for the thorough review of our manuscript. We have incorporated the reviewers' comments into a revised manuscript, and we believe the paper is much improved as a result. A response has been provided to each of the reviewer's comments in the section below.

Below is the manuscript author along with contact details and the institutional affiliation.

1. Aston K. McCullough, Ph.D., M.S., M.A., CFP—Laboratory for the Scientific Study of Dance (LAB:SYNC), Center for Cognitive & Brain Health, Department of Physical Therapy, Movement & Rehabilitation Sciences; Northeastern University (a.mccullough@northeastern.edu)
    a. Corresponding author
        360 Huntington Ave
        129 ISEC
        Boston, MA 02109
        Phone: +1 (617) 373-8839

To our knowledge, there are no potential reviewer conflicts to report. The corresponding author stated above agrees with the study findings. Dr. McCullough designed and oversaw the study, acquired the funding, collected all data, analyzed the data, specified and ran all statistical models, and wrote and finalized the manuscript. The author declares there are no conflicts of interest to report. The human subjects research protocol conducted in the study was in adherence with the policies and procedures of, and approved by, the Institutional Review Board at the University of Massachusetts Amherst (Protocol #2070). Laboratory for the Scientific Study of Dance (LAB:SYNC), a National Endowment for the Arts Research Lab, was funded in part by an award from the National Endowment for the Arts (Award #: 1879058-38-C-21). The opinions expressed are those of the author and do not represent the views of the National Endowment for the Arts (NEA) Office of Research & Analysis or the National Endowment for the Arts. The NEA does not guarantee the accuracy or completeness of the information included in this material and is not responsible for any consequences of its use.

Thank you for your time and thoughtful consideration as you review this work.

Sincerely,

Aston K. McCullough, Ph.D., M.S., M.A.
Assistant Professor, Center for Cognitive & Brain Health

Department Of Physical Therapy, Movement & Rehabilitation Sciences
Department of Music
Affiliate Assistant Professor, Khoury College of Computer Sciences
Northeastern University

Review Comments to the Author

Please use the space provided to explain your answers to the questions above. You may also include additional comments for the author, including concerns about dual publication, research ethics, or publication ethics. (Please upload your review as an attachment if it exceeds 20,000 characters)

Reviewer #1: Overall, this manuscript presents a thorough explanation of a well-conducted study. The necessity of this study, through the evidence it aims to add to the ability of free-form dancing to meet exercise intensity criteria to enhance health, is well laid out in some places, but could be further emphasised in others – particularly in relation to the utility/application/implications of the study's key findings.

Two general feedback points are 1) to consider including additional figures and tables. This would help to make large (and at times repetitive) portions of text in the method and results more readily understandable for the reader. Specific examples of where these might be particularly useful are outlined below. 2) throughout the manuscript, the use of acronyms is currently inconsistent, with acronyms and writing out in full used interchangeably for a number of terms. These should be defined on first use and then consistently used thereafter. Most notable is use of MVPA (where appropriate), but other examples include use of VO2/VCO2 in the measures section, and HRmax/HRR (particularly in the discussion).

Specific feedback points to consider are outlined below. The use of line numbers would have aided this review, but the feedback points provided attempt to be as directed as possible.

- **Thank you for the thorough review of this manuscript. The two main points above have been strongly considered, and the manuscript has been revised accordingly. Specifically, additional figures and tables have been included within the methods and results are now more visually interpretable. The re-expansion of any previously defined abbreviations (in the discussion section, specifically) has been revised, so that no abbreviations are re-expanded in the manuscript after first their initial definition. It is hoped that this revision to the abbreviations is most helpful for the readership.**

Abstract
- Objectives – add another sentence as to 'why' this is important to examine

- **Thank you. A sentence has been included that helps point toward the significance of dance as a mode of physical activity.**

- Method – add the specific number of dance bouts undertaken (with range if relevant) rather than stating 'multiple…'

- **Thank you. This sentence has been revised, and the number of bouts has been clarified within the procedures section as well.**

- Method – add the specific variable of interest to the indirect calorimetry description (i.e. calculation of METs)

- **Thank you. We have named and defined METs within the methods section.**

- Methods – include a brief statement of the statistical analyses undertaken to give context to test statistics reported in the results section

- **Yes, thank you for inviting the abstract to make space for this. While the statement about the linear mixed effects model was originally omitted to help with the word count, this important information has been returned to the manuscript.**

- Results – I might suggest removing the first sentence as it is not clear why these data are included here for the moderate bouts, but not for the vigorous. I would instead suggest presenting the last two sentences of this section first, which contain summary data pertaining directly to the key finding

- **Thank you. In the original submission, the first sentence referred to above was intended to ground the readers in the results by offering the intercept for the linear mixed effects models that estimated METs. The second sentence then described key independent variables in the linear mixed effects model and noted the effect of engaging in dance at a self-determined vigorous intensity. The order of the results about the absolute and relative intensities has been revised, as requested, and punction has been revised in the statements about METs to help better connect all related results.**

- Conclusions – consider adding a final sentence stating the possible implications/applications of the key findings (adding the 'why' element more clearly again)

- **Thank you for the invitation to situate the findings more deeply into the existing literature. The conclusions have been revised to say more about the implications of the study findings—that is to say that, engaging in free-form dance can be incorporated into one's weekly PA routine toward accumulating the recommended >=150 minutes of moderate PA.**

Introduction

- While the focus is clearly outlined as being concerned with addressing a gap in the existing literature specifically surrounding the intensity of free-form dance activity, it would be pertinent to also consider the durational component of physical activity recommendations. E.g. what are the current recommendations for duration of sustained MVPA?

- **Thank you for this very important public health-oriented question. The latest recommendations available from the 2018 Physical Activity Guidelines for Americans have**

been included as part of the revision. With this significant revision, the importance of engaging in moderate-to-vigorous physical activity bouts of any duration is cited and underscored. Thus, the significance of the study with respect to engaging in short bouts of free-form dance is further highlighted by way of responding to this important comment.

- Given the use of music as a manipulated variable within the design, it would be pertinent to also provide a summary of existing evidence on the possible influence of music (preference, tempo, volume, etc.) on self-determined exercise intensity (measured and perceived).

- **The introduction has been revised to further highlight the significance of music within the study design.**

Methods

Sample
- Exclusion criteria are provided in detail

- **Thank you.**

- Consider adding a flow diagram detailing participant numbers at each stage, i.e. number of individuals who initially responded to the open recruitment advert, number screened out based on stated exclusion criteria, and any other missing or incomplete data sets excluded from analysis

- **Thank you for this suggestion. The methods section now provides detailed descriptions of the number of participants who provided informed consent, in addition to the number that withdrew from the study, along with reasons for participant withdrawal from the study. In the original manuscript, focus was placed exclusively on the participants who returned to complete the free-form dance session; in the revision a detailed description has been interleaved into the text to describe the participant flow through the study activities after obtaining informed consent.**

Procedures
- Refer to ‘body mass’ instead of ‘weight’ throughout

- **Thank you. The manuscript has been updated to use the term ‘body mass’**
    - **doi: 10.1097/NT.0000000000000092**

- Appropriate control measures were implemented, with the condition of participants accounted for. As per the comment above, did any participants not meet these criteria and subsequently miss measurements, have to return on a separate occasion, or need to be excluded from analysis?

- **Please see response to comment above.**

- More information about the music choice process is required: how many pieces were selected? Were these from a set selection (of how many?) or completely open for the individual to choose?

- **Thank you very much. Importantly, this revision clarifies that it was completely open for every individual to choose each piece of music from their own personal music library.**

- More information about the dance bouts is required: how many 5-minute bouts did they complete in total and was this consistent across participants (or a range)? Were the moderate and vigorous bouts specifically instructed (i.e. "try to reach a moderate intensity for this bout")? If not, how were these differentiated for analysis? Were moderate bouts always performed first or were they randomised? A diagram of the experimental set up would be useful to give the reader a clearer understanding of how this was practically managed.

- **Thank you; a diagram has been included as suggested (Figure 1). We have also clarified that the self-determined intensities across moderate and vigorous intensity bouts were respectively determined by each individual participant. The number of bouts has also been underscored as being four.**

Measures
- Good detail is provided

- **Thank you.**

- Was the participant who didn't complete the REE measure excluded from all analyses or just these data? How was this managed? (This should be added to the sample size diagram mentioned above)

- **The participant without REE was not included in reports for any test that used REE (i.e., WR:RMR and descriptive statistics of REE).**
- **The original version of the manuscript underscored the sample size in each figure and table for the readers. The lower sample size for REE and WR:RMR were respectively indicated in the original manuscript within the methods and results section.**
- **Now, in responding to the suggestion to translate key statistical results into a table, the sample size has been underscored across each place results are reported to ensure readers can confirm the sample size for each test.**
- **Therefore, the associated sample size is clearly presented to the readership for every statistical test that was conducted.**

Statistical analyses
- Good detail is provided and the inclusion of justification for tests chosen is good to see

- Thank you for your careful review.

Results
- It is not clear where missing data is accounted for in the reported n (this should also be a small case n for sample size)

- **Thank you for identifying the secondary capitalized 'N' typo -- the case of the sample size indicator (n) has been appropriately revised.**
- **Please note response to question above regarding sample size and missing cases**

- Consider reporting key values in table format to allow easier comparison across conditions than is currently possible within the large amount of text presented, e.g., mean (95% CI) values for different conditions and TOST results.

- Thank you for inviting this revision to include additional key summary tables and figures. The revision now includes a summary table of the absolute and relative intensities for each condition, along with 95% CI. The revision also includes a typical TOST figure that allows for visual comparison of the 95% CI across conditions.

Discussion

- The key findings are well discussed with reference to the exiting evidence base

- **Thank you.**

- As mentioned under the introduction, the durational component for physical activity guidelines is missing here. Would the intensities seen during the five-minute bouts be sustained by participants over a longer duration? What would the mean METs and/or overall RPE be over say 20 min? This should perhaps be acknowledged as a limitation of the present study and a question to examine in future research.

- **Thank you again for this comment that ties the introduction and discussion sections to the current PA recommendations for adults. The revised manuscript discusses the current recommendation that >=150 minutes of moderate intensity PA should be accumulated weekly in bouts of any duration, per the latest recommendations presented in the 2018 Physical Activity Guidelines for Americans.**
