# Supplementary material for: Absolute and relative intensities of solo, free-form dancing in adults: A pilot study
Source: PLoS One. 2024 Nov 18;19(11):e0313144. doi: 10.1371/journal.pone.0313144 (PMC11573202; doi:10.1371/journal.pone.0313144)
Supplement: Attachment — Submitted filename: PLOS_ONE_CoverLetter_AbsRelRevision_09192024.pdf [file pone.0313144.s003.pdf]

Northeastern
Bouvé College of Health Sciences

Emily Chenette, PhD
Editor-in-Chief, *PLOS ONE*
PLOS
1265 Battery Street, Suite 200
San Francisco, CA 94111
United States

19 September 2024

Dear Dr. Chenette:

On behalf of my research group, I am pleased to resubmit the manuscript titled "Absolute and relative intensities of solo, free-form dancing in adults: A pilot study" to be considered for publication as an original research article in *PLOS ONE*. Thank you for the thorough review of our manuscript. We have incorporated the most recent round of comments from the reviewers' into a revised manuscript, and we believe the paper is much improved as a result. A response has been provided to each of the reviewer's comments in the section below.

Below is the manuscript author along with contact details and the institutional affiliation.

1. Aston K. McCullough, Ph.D., M.S., M.A., CFP—Laboratory for the Scientific Study of Dance (LAB:SYNC), Center for Cognitive & Brain Health, Department of Physical Therapy, Movement & Rehabilitation Sciences; Northeastern University (a.mccullough@northeastern.edu)
   a. Corresponding author
      360 Huntington Ave
      129 ISEC
      Boston, MA 02109
      Phone: +1 (617) 373-8839

To our knowledge, there are no potential reviewer conflicts to report. The corresponding author stated above agrees with the study findings. Dr. McCullough designed and oversaw the study, acquired the funding, collected all data, analyzed the data, specified and ran all statistical models, and wrote and finalized the manuscript. The author declares there are no conflicts of interest to report. The human subjects research protocol conducted in the study was in adherence with the policies and procedures of, and approved by, the Institutional Review Board at the University of Massachusetts Amherst (Protocol #2070). Laboratory for the Scientific Study of Dance (LAB:SYNC), a National Endowment for the Arts Research Lab, was funded in part by an award from the National Endowment for the Arts (Award #: 1879058-38-C-21). The opinions expressed are those of the author and do not represent the views of the National Endowment for the Arts (NEA) Office of Research & Analysis or the National Endowment for the Arts. The NEA does not guarantee the accuracy or completeness of the information included in this material and is not responsible for any consequences of its use.

Thank you for your time and thoughtful consideration as you review this work.

Sincerely,

Aston K. McCullough, Ph.D., M.S., M.A.
Assistant Professor, Center for Cognitive & Brain Health
Department Of Physical Therapy, Movement & Rehabilitation Sciences

Department of Music
Affiliate Assistant Professor, Khoury College of Computer Sciences
Northeastern University

Review Comments to the Author

Please use the space provided to explain your answers to the questions above. You may also include additional comments for the author, including concerns about dual publication, research ethics, or publication ethics. (Please upload your review as an attachment if it exceeds 20,000 characters)

Reviewer #1: Thank you for the detail paid in responding to comments and submitting the revised version of the manuscript. I believe this is a detailed account of a well conducted and technically sound study, that is suitable for publication.

- **Many thanks again for your thoughtful review of this manuscript. The revisions that were suggested strengthened the manuscript.**

Reviewer #2: The Manuscript presents a pilot study characterizing the absolute and relative physical activity (PA) intensities of solo, free-form dancing at self-determined moderate and vigorous intensities in adults. The examination was set to investigate if dancing however one wishes at such perceived intensities may support adults in accumulating the recommended weekly dose of equal or more than 150 minutes of moderate intensity PA. The BMI and use of music background for activity were considered.

The Manuscript provided data that supports the conclusions: thorough presentation of data collection and analysis.
The research was conducted rigorously, with appropriate controls. The procedures were planned with great care in a detailed manner including the criteria determining the choice of participants of the examined group as well as conditions in which examination took place. Absolute intensity was measured during free-form dancing bouts using indirect calorimetry (metabolic equivalents; METs = $\dot{V}$ O2 ml·kg-1·min-1/3.5). Relative intensity was measured by ratings of perceived exertion (Borg scale) and heart rate. Linear mixed effects models were used assess the relationship between absolute and relative intensity metrics and model covariates. Body Mass Index (BMI) was measured using a calibrated Seca 286 stadiometer and scale (Seca, Hamburg, Germany). Sociodemographic characteristics of the group were collected by self-report questionnaires administered to participants via REDCap – they also included self-reported data of total years of dance training experience. Music Information Retrieval Toolbox was used to extract the tempo of each self-selected musical piece that participants chose to accompany their dance bouts.

Ethical approval for the study procedures and methods was granted. The human subjects research protocol conducted in the study was in adherence with the policies and procedures of, and approved by, the Institutional Review Board at the University of Massachusetts Amherst (Protocol #2070). Written and verbal consent was gained from participants prior to study participation.

The conclusions were drawn appropriately based on the data presented and clearly referred to the hypotheses formulated at the beginning of the research process. The statistical analysis was performed appropriately and rigorously. Data were analyzed in MATLAB (The Mathworks Inc. MATLAB R2022a.). To test for equivalence between music tempi in the self-selected moderate and vigorous intensity bouts, a non-parametric two one-sided tests of equivalence (TOST) model was implemented. Linear mixed effects models (Snijders, T. A. B. & Bosker, R. J., 2012) were used to estimate associations between the relative and absolute intensity of free-form dance bouts, using each respective measure of physical activity intensity, self-determined intensity (intensity condition), and sociodemographic (age), anthropometric (BMI), and environmental factors (presence of music).

The authors successfully proved that adults were able to modulate the PA intensity of free-form dancing at will between self-determined moderate and vigorous intensity dance bouts. The presence of music was positively associated with the absolute PA intensity of free-form dancing. When characterized using HRR, engaging in free-form dance at self-determined moderate-to-vigorous PA intensities provided a sufficient stimulus for all adults to reach a moderate PA intensity, which provides evidence that dancing however one wishes at such perceived intensities may support adults in accumulating the recommended weekly dose of equal or more than 150 minutes of moderate intensity PA.

All data underlying the findings in The Manuscript is fully available. It can be found within the manuscript and its Supporting Information files.

The Manuscript is presented in an intelligible fashion and written in standard English with a good proficiency.

- **Thank you kindly for your rigorous review of the manuscript. The specificity and generous attention to detail are greatly appreciated.**

Comments:

- "Dance" or "Free Dance" would be suggested to add in the Key Word section, as it is main activity examined in the study.

- **Thank you very much for this suggestion. Dance has been included as a keyword.**

There is some inconsistency in usage of letter "s" or "z" in the word "analyze". Unification throughout The Manuscript would be advised. There are also minor typos to correct, for example: "tempi" instead "tempo".

- **Thank you for the suggestion to unify the style of spelling with reference to "analyses" (noun, plural) and "analyze" (verb). In the original manuscript "analyze" (verb) was used (U.S. English) and "analyses" (noun, plural) was used, which appear to be unified for this (U.S. English) reader.**

  The manuscript has been carefully reviewed for any possible typos, and where tempi is used the plural form of tempo is intended.

- The tables and charts could be formatted to fit into text.

- **We believe that here the reviewer is kindly suggesting that the revision should clearly indicate the intended placement of tables and figures in the text. We have made updates throughout the manuscript to indicate how we envisioned the tables/figures might possibly be formatted to fit into the final text. Thank you.**

- Since The Manuscript refers to “Free Dance” and “Improvisation”, extended characteristics of these activities in the theoretical introduction would be advised

- **In the original manuscript, free-form dance was operationalized by inviting participants to dance as they wanted. In this revised manuscript, the introduction now expands on statements from the prior version to help connect the theoretical introduction to the procedures detailed in the methods.**
    - **Introduction (pg. 3):**
        - “To dance as one wants, or feels inspired at any moment, is fundamental to engaging in free-form dance behavior—ostensibly, one can choose to dance however one wishes, most wherever one wants, and with few resources.”
        - “Free-form dance behavior, in contrast to codified dance styles, is highly heterogenous in its expression given the individualized, idiosyncratic characteristics of improvisational dancing and its general lack of rehearsed motor sequences.”
    - Methods (pg. 5)
        - “Participants were invited to “dance in their usual fashion” both with and without music for five-minute bouts at respectively self-determined moderate and vigorous activity intensities (see Figure 1).”
        - “Before participants began dancing, the researcher informed each participant that they ‘would not really be watching’ the participant dance and that they would be ‘mostly focused on the data’ during the dance bouts.”

- **The theoretical introduction leverages the available, relevant peer-reviewed literature without including the opinion of the author to characterize free-form dance; additional research is urgently needed on the kinematic characteristics of free-form dance.**

  Thank you for this question that further reveals the gap in the literature which this study seeks to begin to fill—that is, there is a dearth of peer-reviewed research that characterizes free-form dance to date. With the hopeful publication of this paper, it will be the first in a series of manuscripts that seek to comprehensively investigate free-form dance.

- Although The Manuscript presents a pilot study, small control group could be considered for reference.

Thank you for this important point. In writing this paper, the manuscript's length and content became substantial and complete. Data on self-paced walking (a control condition) in comparison to free-form dancing were saved for further analysis and future publication, which will offer an opportunity to report a detailed description of those methods, results, and further discussion. The data on WR:RMR also use a resting measure (RMR) as a reference against which PA intensity during dance was normalized.
